# Exploring the role of racial/ethnic patient–physician concordance in optimizing health and patient outcomes among black populations in the United States: an integrative systematic review

**DOI:** 10.3389/fpubh.2026.1838171

**Published:** 2026-06-24

**Authors:** Christopher Washington, Tanisha Maitre, Nicholas Sobowale, Bryant Lewis

**Affiliations:** 1 Lewis Katz School of Medicine, Temple University Hospital, Philadelphia, PA, United States; 2 Cancer Prevention and Control Program, Fox Chase Cancer Center, Philadelphia, PA, United States

**Keywords:** African immigrants, Afro-Caribbean immigrants, cultural sensitivity, Foundational Black Americans, race/ethnic concordance, social determinants of health

## Abstract

**Introduction:**

Black populations in the United States experience disproportionately high burdens of morbidity and mortality across numerous health outcomes. However, substantial heterogeneity exists within these populations with respect to migration history, socioeconomic context, social determinants of health, and healthcare experiences. For analytic clarity, this review distinguishes Foundational Black Americans (FBAs)—defined here as U.S.-born Black Americans descendant from US chattel slavery—from Black immigrant populations, including Afro-Caribbean and African immigrants. Disaggregation of broad racial categories is increasingly recognized as an important epidemiologic strategy for improving measurement precision and identifying population-specific determinants of health.

**Methods:**

This integrative systematic review examined whether physician–patient racial/ethnic concordance is associated with healthcare communication, trust, satisfaction, shared decision-making, healthcare utilization, adherence, and clinical outcomes among Black populations in the United States. Structured searches of PubMed and Web of Science identified studies published between 2015 and 2026. Following screening and duplicate removal, 31 studies met inclusion criteria.

**Results:**

Communication and trust were the most frequently examined domains (14/31 studies), followed by patient satisfaction and engagement (9/31), clinical outcomes (8/31), healthcare utilization and adherence (6/31), and workforce representation (3/31). Across the literature, most studies reported at least one favorable association between racial/ethnic concordance and communication quality, patient trust, satisfaction, engagement, healthcare utilization, adherence, or selected clinical outcomes. Evidence was strongest for interpersonal and patient-experience outcomes, whereas findings for clinical outcomes were more heterogeneous and context dependent. Most included studies were observational or cross-sectional, limiting causal inference. Substantial variation in study design, exposure definitions, and outcome measurement further limited comparability across studies.

**Discussion:**

Overall, physician–patient racial/ethnic concordance appears to function as one meaningful interpersonal and structural factor associated with healthcare experiences among Black populations. Workforce diversification and culturally responsive care may represent complementary strategies for improving patient engagement and reducing disparities. Future research should incorporate more refined subgroup characterization within Black populations to better understand heterogeneity in concordance-related effects.

## 1 Introduction

### 1.1 Article organization

This review is organized to clarify why heterogeneity within Black populations in the United States may be relevant to the interpretation of healthcare disparities and physician–patient racial/ethnic concordance research. It argues that medical institutions should have the capacity to differentiate among Black subgroups when appropriate for research, care delivery, and intervention design, while also acknowledging the broader cultural and historical factors that shape the experiences of Foundational Black Americans (FBAs). The review first examines major health disparities affecting Black populations in the United States, then discusses subgroup heterogeneity within Black populations, and finally synthesizes contemporary evidence examining whether racial/ethnic concordance between patients and physicians is associated with healthcare experiences and outcomes.

Although prior reviews have examined racial/ethnic concordance and patient outcomes, fewer studies have synthesized recent evidence across multiple healthcare domains while also considering how heterogeneity within Black populations may complicate interpretation of aggregated racial categories (68, 69, 72–74, 76, 77, 81, 88). In addition, newer studies examining concordance in mental health, addiction treatment, telehealth, surgical care, and postpartum care have not been comprehensively integrated within prior reviews (82, 90, 91).

The objective of this review is to systematically examine whether physician–patient racial/ethnic concordance is associated with improvements in healthcare experiences, engagement, and clinical outcomes among Black populations in the United States, while also discussing how heterogeneity within Black populations may influence interpretation of concordance-related findings.

Unlike prior reviews that focused primarily on physician–patient racial/ethnic concordance broadly or within specific clinical domains, this review synthesizes contemporary evidence published between 2015 and 2026 across communication, trust, satisfaction, healthcare utilization, adherence, clinical outcomes, addiction treatment, surgery, maternal health, and workforce representation. Additionally, the review explicitly considers how heterogeneity within Black populations may influence interpretation of concordance-related findings and identifies important gaps in the current literature regarding subgroup-specific healthcare experiences.

To our knowledge, this is among the first reviews to integrate contemporary concordance evidence across communication, trust, satisfaction, healthcare utilization, adherence, surgery, addiction treatment, maternal health, and workforce representation while simultaneously examining how aggregation of heterogeneous Black populations may influence interpretation of concordance-related findings.

### 1.2 The predominant health disparities affecting Black populations in the United States

Black populations in the United States continue to experience substantial disparities in morbidity and mortality across numerous health outcomes (1–6). National surveillance data demonstrate disproportionately elevated rates of hypertension, diabetes, stroke, chronic kidney disease, and cardiovascular mortality among Black populations relative to White populations (2–6). These disparities often emerge earlier in the life course and are associated with more severe disease trajectories.

For example, Black adults experience higher rates of hypertension and cardiovascular disease mortality than White adults, with Black Americans demonstrating significantly increased likelihoods of stroke-related mortality, cardiovascular mortality, and end-stage renal disease (3, 6). Black patients are also more likely to experience resistant hypertension and challenges related to medication adherence (5, 7, 8).

Several studies suggest that healthcare communication, patient engagement, trust, and collaborative provider–patient relationships may influence adherence and chronic disease management outcomes (9–13). Shared decision-making, patient-centered communication, and provider responsiveness have all been associated with improved treatment engagement and adherence behaviors (9–11). However, disparities in patient–provider communication and trust may reduce the effectiveness of these approaches among racially minoritized populations (13, 14).

Collectively, these findings highlight the importance of examining not only biological disease burden, but also the social, cultural, and structural determinants shaping healthcare experiences among Black populations in the United States.

### 1.3 Recognizing heterogeneity within Black populations in the United States

In epidemiologic and health disparities research, disaggregation of broad racial and ethnic categories is recognized as an important strategy for improving measurement precision and identifying at-risk populations. Aggregated racial labels may obscure meaningful within-group heterogeneity relevant to health outcomes, healthcare experiences, and responses to intervention. In the United States, the term “African American” is commonly used to describe individuals of African descent; however, this designation encompasses heterogeneous populations that include U.S.-born Black individuals with multigenerational roots as well as more recent immigrants from the Caribbean and Africa (70, 71, 75, 83, 84, 89). These groups may differ in migration history, socioeconomic context, cultural practices, and exposure to U.S.-specific structural determinants of health.

To improve analytic clarity, this review uses the term “Foundational Black Americans (FBAs)” to refer to U.S.-born Black individuals with multigenerational ancestry rooted in populations historically subjected to chattel slavery within the United States. This terminology is used as an analytic construct to distinguish this population from Black immigrant subgroups, including Afro-Caribbean and African immigrants, while recognizing that such distinctions are not consistently operationalized within national datasets. Where direct lineage measures are unavailable, commonly used proxies such as nativity and parental birthplace may be used to approximate subgroup differences.

Prior studies suggest that important differences exist across Black subgroups in the United States. Commodore-Mensah et al. (15), using National Health Interview Survey (NHIS) data, reported higher prevalence of hypertension and diabetes among FBAs relative to African immigrants and Afro-Caribbean immigrants. Similarly, AHA-affiliated analyses reported meaningful variation in cardiovascular risk profiles across Black subgroups, with U.S.-born Black adults demonstrating higher hypertension and obesity prevalence than African immigrant subgroups (16–19). Differences have also been observed in cancer mortality patterns, with Pinheiro et al. (20) reporting that U.S.-born Black Americans had the highest all-sites-combined cancer mortality rates among the Black subgroups analyzed.

Collectively, these findings suggest that broad racial aggregation may obscure clinically and socially meaningful heterogeneity within Black populations in the United States. However, it is important to note that FBA status is not routinely measured in most healthcare datasets. As such, subgroup-related interpretations should be understood within the context of available proxy measures and the broader limitations of population-level racial classification.

### 1.4 Historical context and social determinants of health

Differences in health outcomes across Black subgroups may partially reflect variation in socioeconomic conditions, migration history, and exposure to structural determinants of health (79, 86). Prior studies have demonstrated differences in educational attainment, chronic disease risk factors, healthcare experiences across Black populations in the United States (15–23). Educational attainment among African immigrants, for example, has been reported to exceed that of several U.S.-born populations, including FBAs (24–26, 87). Other studies suggest that immigrant status may confer partial protection against certain adverse educational and health-related outcomes observed among U.S.-born Black populations (21, 22).

Historical and structural determinants of health remain important when interpreting healthcare experiences among Black populations in the United States (67, 78, 80, 85, 92). Prior research has documented the long-term effects of slavery, segregation, healthcare discrimination, and structural racism on education, income, neighborhood conditions, healthcare access, and institutional trust (23, 27–29).

These factors may influence how patients engage with healthcare systems, communicate with clinicians, evaluate provider recommendations, and develop trust in medical institutions. Because communication, trust, and engagement are amongst the most commonly proposed mechanisms underlying concordance-related effects, subgroup differences in historical experience and social determinants of health may be relevant when interpreting concordance findings.

Recognizing heterogeneity within Black populations may therefore improve epidemiologic precision, strengthen interpretation of healthcare disparities, and support development of more culturally responsive approaches to care (30). At the same time, most existing studies examining racial/ethnic concordance do not directly differentiate among Black subgroups, highlighting an important limitation within the current evidence base and an area for future research.

## 2 Methodology

An integrative systematic review was conducted to examine whether physician–patient racial/ethnic concordance is associated with healthcare experiences and outcomes among Black populations in the United States. The review synthesized evidence across heterogeneous study designs, with particular attention to trust, communication, patient satisfaction, healthcare utilization, shared decision-making, adherence, clinical outcomes, and workforce-related access to concordant care.

### 2.1 Data sources and search strategy

A structured literature search was conducted in **PubMed** and **Web of Science**. Both searches were performed on **May 22, 2026**, and were limited to articles published between **January 1, 2015 and May 22, 2026**. This timeframe was selected to emphasize contemporary evidence generated during a period of increased attention to health equity, patient-centered care, workforce diversity, and racial/ethnic concordance in healthcare research.

The following search strategy was applied to both databases:

(“racial concordance”[Title/Abstract] OR “ethnic concordance”[Title/Abstract] OR “race concordance”[Title/Abstract] OR “patient physician concordance”[Title/Abstract] OR “patient provider concordance”[Title/Abstract]) AND (“Black patients”[Title/Abstract] OR “African American”[Title/Abstract] OR minority[Title/Abstract]) AND (trust[Title/Abstract] OR communication[Title/Abstract] OR satisfaction[Title/Abstract] OR adherence[Title/Abstract] OR utilization[Title/Abstract] OR outcomes[Title/Abstract] OR mortality[Title/Abstract] OR “shared decision making”[Title/Abstract]).

The PubMed search yielded **54 records**, of which **25 studies** met inclusion criteria. The Web of Science search yielded **12 records**, of which **6 non-duplicative studies** met inclusion criteria. The final synthesis therefore included **31 studies**. The full search strategy, database-specific results, and screening outcomes are provided in Supplementary Table S1.

A review protocol was not prospectively registered, and no publicly accessible protocol document is available.

### 2.2 Eligibility criteria

Studies were included if they met the following criteria:

1. published between 2015 and 2026;
2. examined physician–patient or provider–patient racial/ethnic concordance, discordance, or closely related mechanisms;
3. addressed healthcare experiences, communication, trust, satisfaction, shared decision-making, adherence, utilization, clinical outcomes, or workforce-related access to concordant care; and
4. focused on U.S.-based populations or healthcare contexts relevant to Black populations in the United States.

Studies were excluded if they:

1. fell outside the specified publication window;
2. did not address racial/ethnic concordance, discordance, or a directly relevant concordance-related mechanism;
3. focused primarily on non-U.S. populations;
4. focused primarily on language concordance without a racial/ethnic concordance component;
5. were conference abstracts without sufficient methodological detail; or
6. addressed broad racial disparities without direct relevance to patient–provider concordance, communication, trust, healthcare utilization, or clinical outcomes.

### 2.3 PICOS framework

The review question was structured using a PICOS framework.

**Population:** Black patients, racially/ethnically minoritized patients, clinicians, or healthcare systems in U.S.-based settings.

**Intervention/Exposure:** Physician–patient or provider–patient racial/ethnic concordance, racial/ethnic discordance, or related mechanisms such as workforce representation, communication dynamics, and culturally concordant care.

**Comparator:** Racially/ethnically discordant care, non-concordant care, or absence of concordance-related exposure.

**Outcomes:** Patient trust, communication quality, satisfaction, shared decision-making, adherence, healthcare utilization, healthcare expenditures, clinical outcomes, mortality, and workforce-related access to concordant care.

**Study Designs:** Quantitative, qualitative, mixed-methods, observational, interventional, systematic review, scoping review, and conceptually relevant peer-reviewed studies.

### 2.4 Study selection

Study selection followed a structured, sequential process. Records were first screened by title and abstract for relevance to the review objective. Articles appearing to meet inclusion criteria were then assessed through full-text review.

**Figure 1:**
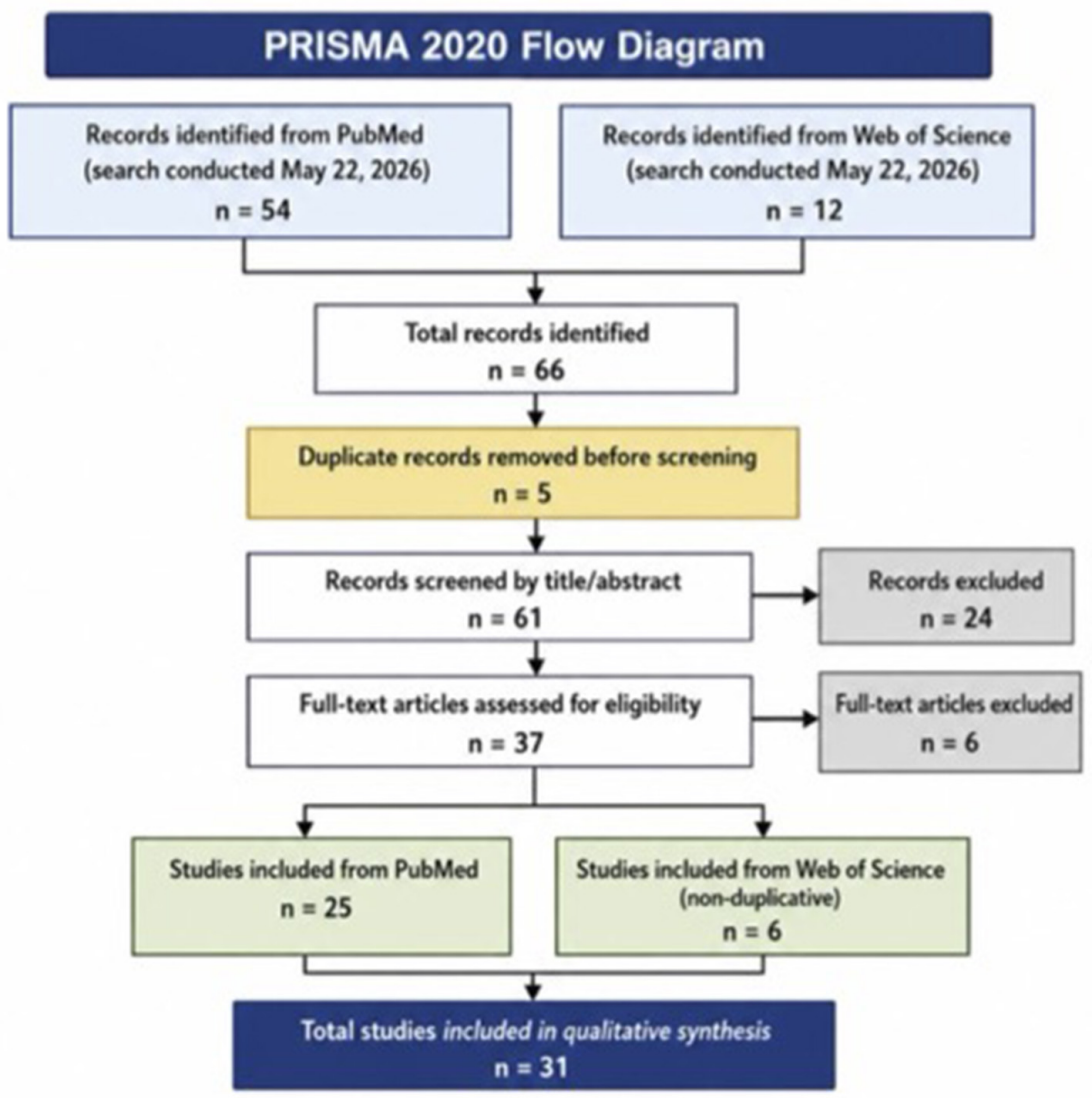
PRISMA 2020 flow diagram of study identification, screening, duplicate removal, and study inclusion for the present integrative review. Literature searches were conducted in PubMed and Web of Science on May 22, 2026, and limited to publications from January 1, 2015 to May 22, 2026. PubMed yielded 54 records and Web of Science yielded 12 records. After removal of 5 duplicate records, 61 records were screened by title and abstract. Thirty-seven full-text articles were assessed for eligibility, and 6 were excluded. Twenty-five studies were included from PubMed and 6 non-duplicative studies were included from Web of Science, resulting in 31 total studies included in the final qualitative synthesis.

Duplicate records identified across PubMed and Web of Science were removed prior to final inclusion. The PubMed search produced 54 records, with 25 retained for synthesis. The Web of Science search produced 12 records, with 6 non-duplicative studies retained. The final sample comprised 31 included studies, as shown in Figure 1.

Study screening was conducted by two reviewers. Discrepancies were resolved through discussion and consensus.

### 2.5 Data extraction

Data extraction was performed using a standardized framework. For each included study, the following variables were extracted: first author, publication year, study population, clinical or healthcare setting, study design, concordance-related exposure, key outcomes, principal findings, and whether the findings supported an association between racial/ethnic concordance and improved healthcare experiences or outcomes.

#### 2.5.1 Classification of included literature

The included literature was classified into two categories for synthesis purposes: **Primary Evidence Studies (*n*** **=** **22)** and **Contextual/Conceptual Articles (*n* =** **9)**. Primary Evidence Studies included quantitative, qualitative, mixed-methods, observational, experimental, and workforce analyses that contributed original empirical findings. Contextual/Conceptual Articles included systematic reviews, scoping reviews, methodological papers, commentaries, and conceptual articles that provided interpretive, theoretical, or contextual information relevant to racial/ethnic concordance, communication, implicit bias, health equity, and workforce representation.

Conceptual and contextual articles were used to inform interpretation of the evidence base and identify broader themes; however, they were not weighted equally with primary empirical studies when drawing conclusions regarding concordance-related associations and healthcare outcomes.

The inclusion of contextual and conceptual articles was consistent with the integrative review methodology employed in this study. Integrative reviews permit the inclusion of diverse forms of evidence to facilitate comprehensive understanding of complex phenomena that may not be fully captured through empirical outcome studies alone. Accordingly, conceptual and contextual articles were used to identify theoretical mechanisms, interpret broader patterns within the literature, and contextualize empirical findings, but were not treated as equivalent evidence for outcome-based conclusions.

Extracted outcomes included patient–physician trust, communication quality, patient satisfaction, shared decision-making, healthcare utilization, adherence, healthcare expenditures, clinical outcomes, mortality, and workforce-related indicators of access to concordant care.

Extracted study characteristics are presented in Table 1.

**Table 1:** Detailed characteristics and principal findings of included studies ← expanded study characteristics table.

Study (Reference)	Design	Sample size	Population	Setting	Outcome(s)	Principal finding
Walker et al. (44)	Primary empirical; observational/cross-sectional	3,672 unique patient encounters	Primary care patients	Primary care	Satisfaction; racial/ethnic and sex concordance	Examined whether racial/ethnic and sex concordance were associated with primary care satisfaction scores.
Suleiman et al. (45)	Primary empirical; observational	314 patients	Patients evaluated for total knee arthroplasty	Orthopedic surgery	TKA recommendation	Patient–physician racial concordance increased likelihood of total knee arthroplasty recommendation.
Poma (51)	Contextual/conceptual article	N/A	N/A	General medical care	Racial/ethnic concordance	Discussed implications of race/ethnicity concordance between patients and physicians.
Gross and Barry (65)	Contextual/conceptual article	N/A	Black/African American patients	Health equity context	Concordance; health equity	Framed patient-provider racial concordance as relevant to Black health equity.
Shen et al. (66)	Systematic review	40 included articles	Black patients in healthcare contexts	Patient–physician communication literature	Communication quality; information-giving; participation; decision-making	Black patients experienced poorer communication than White patients; racial concordance was associated with better communication across several domains, though findings were heterogeneous.
Anderson et al. (31)	Primary empirical; simulated clinical interaction study	107 adult patients and 13 physicians	Racial/ethnic minority participants	Simulated clinical encounters	Pain outcomes	Clinician–patient racial/ethnic concordance influenced pain-related outcomes.
Jetty et al. (52)	Primary empirical; observational database analysis	50,626 adults	Racial/ethnic minority patients	Healthcare utilization/expenditure data	Healthcare use; expenditures	Patient–physician racial concordance was associated with improved healthcare use and lower expenditures among minority populations.
Sweeney et al. (48)	Primary empirical; observational survey analysis	Medical Expenditure Panel Survey years 2002–2012	Patients with usual source of care providers	Medical Expenditure Panel Survey, 2002–2012	Communication quality	Communication ratings were more strongly associated with patient race/ethnicity than concordance after adjustment.
Thomas et al. (32)	Primary empirical; randomized clinical trial	343 patients	Black patients at risk for sudden cardiac arrest	Cardiology decision-making	Shared decision-making	Tested an intervention to facilitate shared decision-making among Black patients at risk for sudden cardiac arrest.
Beaugard et al. (33)	Scoping review	N/A	Black patients in addiction treatment	Addiction treatment literature	Treatment engagement; treatment experience	Found limited but important evidence suggesting racial concordance may influence engagement and treatment experiences for Black patients in addiction treatment.
Kindratt et al. (53)	Primary empirical; observational MEPS analysis	327 women	Non-pregnant women of childbearing age with diabetes mellitus	Medical Expenditure Panel Survey, 2010–2019	Diabetes care; concordance	Examined influence of racial/ethnic and gender concordance on care among women with diabetes.
Moore et al. (54)	Primary empirical; qualitative study	47 caregivers	Black patients	Healthcare experiences	Provider preference; trust; communication	Black patients reported preference for racially concordant providers due to trust, comfort, communication, and perceived understanding.
Gorbatenko-Roth et al. (34)	Primary empirical; survey study	19 patients	Black dermatology patients	Dermatology	Perception of care	Assessed Black patients' perceptions of dermatology care.
Johnson (46)	Contextual/conceptual article	N/A	Black mothers, children, and infants	Maternal and infant health	Racial concordance; maternal/infant protection	Framed racial concordance as a potential strategy to protect Black mothers, children, and infants.
Charlot et al. (55)	Primary empirical; observational study	1,257 women	Patients with cancer screening abnormalities	Cancer navigation/follow-up care	Follow-up after abnormal screening	Patient-navigator race and language concordance influenced care after cancer screening abnormalities.
Scheid and Smith (49)	Primary empirical; observational study	2,815 adults	Women of low socioeconomic status	Women's health	Trust	Examined whether physician–patient concordance was associated with greater trust.
Saha and Beach (35)	Primary empirical; randomized video-vignette experiment	238 patients	Adult participants viewing physician vignettes	Experimental vignette setting	Decision-making; physician ratings	Physician race influenced patient decision-making and ratings of physicians.
Ma et al. (36)	Primary empirical; observational MEPS analysis	25,045 respondents	Patients in MEPS	Medical Expenditure Panel Survey	Provider visits	Patient-provider race/ethnicity concordance was associated with provider visit patterns.
Assari (47)	Primary empirical; observational analysis	336 individuals	Patients in racially concordant and discordant interactions	Patient–physician communication	Communication satisfaction	Psychosocial determinants of communication satisfaction differed across concordant and discordant interactions.
Shannon et al. (37)	Primary empirical; observational study	6,004 patients	Older adults operated on by California licensed surgeons	Surgery	Surgical outcomes	Patient-surgeon racial/ethnic concordance was associated with surgical outcomes among older adults.
Crawford et al. (56)	Primary empirical; pilot study	437 patients	Hospitalized patients	Hospital medicine	Hospitalist performance assessment	Racial and gender concordance influenced patient assessment of hospitalist performance.
Ma et al. (38)	Primary empirical; observational study	238,355 observations	Patients in healthcare relationships	Medication adherence/chronic disease care	Medication adherence; patient-provider relationship	Examined whether racial/ethnic concordance mattered for medication adherence and patient-provider relationship measures.
Rathert et al. (50)	Primary empirical; observational/comparative study	1,598 patients	Black, White, and Hispanic/Latino patients	Healthcare management/patient experience	Therapeutic connection	Patient-provider therapeutic connection varied by race/ethnicity.
Kim et al. (39)	Primary empirical; workforce database analysis	4,848 residents; 398 Im programs; 205 counties	Physicians in U.S. internal medicine residency programs	Graduate medical education	Workforce representation	Found variation in community racial/ethnic representation among physicians in internal medicine residency programs.
Kirksey et al. (58)	Primary empirical; workforce analysis	44 surgeons	Vascular surgeons	Vascular surgery workforce	Workforce diversity	Documented racial diversity patterns and underrepresentation of Black vascular surgeons.
Holm et al. (59)	Primary empirical; observational All of Us analysis	154,803 participants	All of Us participants, 2017–2023	National research cohort	Provider similarity; concordance preferences	Many participants considered provider similarity important; preference was stronger among racially/ethnically minoritized groups.
Hagiwara et al. (60)	Contextual/conceptual article	N/A	N/A	Provider communication training	Implicit bias; communication behaviors	Connected provider implicit racial bias to communication behaviors and disparities; proposed future directions for communication training.
Lin and Kressin (40)	Primary empirical; secondary cross-sectional survey analysis	1,238	U.S. adults	Nationally representative survey	Treatment decision-making; communication	Racial/ethnic minority respondents received less information about treatment rationale from physicians.
Miller et al. (41)	Mixed-methods systematic review	33 included studies	Minoritized patients across included studies	Physician-patient communication literature	Communication; concordance	Found mixed evidence; most adjusted analyses did not show a consistent relationship between concordance and communication variables.
Hall et al. (42)	Systematic review	15 included studies	Healthcare professionals and trainees	Implicit bias literature	Implicit bias; healthcare outcomes	Found implicit racial/ethnic bias among healthcare professionals and associations with patient-provider interactions and some outcomes.
Hagiwara and Dent (43)	Contextual/conceptual methodological article	N/A	N/A	Racially discordant medical interactions	Communication coding; bias measurement	Argued that existing coding systems may miss nonverbal/paraverbal behaviors important in racially discordant encounters.

### 2.6 Data synthesis

Because included studies varied substantially in design, population, clinical setting, exposure measurement, and outcome definitions, quantitative pooling and meta-analysis were not appropriate. Findings were therefore synthesized narratively.

Studies were grouped into thematic domains: communication and trust; patient satisfaction and preference; healthcare utilization, adherence, and costs; clinical outcomes; and workforce representation or structural access to concordant care. Evidence was interpreted with attention to consistency of findings, study design, population relevance, and whether results directly supported concordance as a factor associated with improved healthcare experiences or outcomes.

### 2.7 Risk of bias and certainty of evidence

Given the integrative nature of the review and the inclusion of heterogeneous study designs, a single standardized risk-of-bias tool was not applied. Instead, findings were interpreted in relation to study design, methodological limitations, consistency across studies, and directness of evidence. Formal assessment of reporting bias and certainty of evidence using frameworks such as GRADE was not conducted.

Although formal publication-bias assessment methods are typically applied in meta-analyses, potential reporting bias was considered during interpretation by comparing findings across study designs, populations, and outcome domains.

### 2.8 Study quality assessment

To strengthen interpretation of the included evidence, study quality was assessed using design-appropriate appraisal frameworks. Because the review included heterogeneous study designs, a single quality assessment instrument was not appropriate.

Observational, cross-sectional, cohort, and database studies were evaluated using criteria adapted from the Newcastle–Ottawa Scale (NOS), with consideration given to sample selection, comparability of study groups, outcome assessment, and risk of confounding. Qualitative studies were evaluated using criteria adapted from the Critical Appraisal Skills Programme (CASP) Qualitative Checklist, including clarity of aims, methodological appropriateness, data collection, researcher reflexivity, ethical considerations, and rigor of analysis. Included systematic reviews and scoping reviews were evaluated using criteria adapted from AMSTAR-2, focusing on methodological transparency, comprehensiveness of the literature search, study selection procedures, and synthesis methods.

Studies were classified as high, moderate, or low quality based on overall methodological rigor and relevance to the review question. Because this review synthesized evidence narratively rather than quantitatively, quality assessments were used to contextualize findings and identify limitations within the evidence base rather than to exclude studies from the final synthesis.

A summary of study quality assessments is presented in Table 2.

**Table 2:** Quality assessment of included studies.

Study (Reference)	Study design	Assessment tool	Quality rating
Shen et al. (66)	Systematic Review	AMSTAR-2	Moderate
Anderson et al. (31)	Experimental Study	NOS Adapted Criteria	High
Jetty et al. (52)	Observational Study	NOS Adapted Criteria	High
Sweeney et al. (48)	Observational Study	NOS Adapted Criteria	Moderate
Thomas et al. (32)	Randomized Clinical Trial	NOS Adapted Criteria	High
Beaugard et al. (33)	Scoping Review	AMSTAR-2	Moderate
Kindratt et al. (53)	Cross-Sectional Analysis	NOS Adapted Criteria	Moderate
Moore et al. (54)	Qualitative Study	CASP	High
Gorbatenko-Roth et al. (34)	Cross-Sectional Survey	NOS Adapted Criteria	Moderate
Charlot et al. (55)	Observational Study	NOS Adapted Criteria	High
Scheid and Smith (49)	Observational Study	NOS Adapted Criteria	Moderate
Saha and Beach (35)	Experimental Study	NOS Adapted Criteria	High
Ma et al. (36)	Observational Study	NOS Adapted Criteria	High
Assari (47)	Observational Study	NOS Adapted Criteria	Moderate
Shannon et al. (37)	Observational Study	NOS Adapted Criteria	High
Crawford et al. (56)	Pilot Study	NOS Adapted Criteria	Moderate
Ma et al. (38)	Observational Study	NOS Adapted Criteria	High
Rathert et al. (50)	Observational Study	NOS Adapted Criteria	Moderate
Kim et al. (39)	Workforce Analysis	NOS Adapted Criteria	High
Kirksey et al. (58)	Workforce Analysis	NOS Adapted Criteria	Moderate
Holm et al. (59)	Cross-Sectional Study	NOS Adapted Criteria	High
Hagiwara et al. (60)	Methodological Study	Narrative Appraisal	Moderate
Lin and Kressin (40)	Observational Study	NOS Adapted Criteria	Moderate
Miller et al. (41)	Systematic Review	AMSTAR-2	High
Hall et al. (42)	Systematic Review	AMSTAR-2	High
Hagiwara and Dent (43)	Methodological Study	Narrative Appraisal	Moderate
Walker et al. (44)	Cross-Sectional Study	NOS Adapted Criteria	High
Suleiman et al. (45)	Observational Study	NOS Adapted Criteria	High
Johnson (46)	Commentary	Narrative Appraisal	Low
Poma (51)	Commentary	Narrative Appraisal	Low
Gross and Barry (65)	Commentary	Narrative Appraisal	Low

## 3 Results

### 3.1 Study selection and descriptive characteristics

A total of 66 records were identified through database searching, including 54 records from PubMed and 12 records from Web of Science. After title/abstract screening, full-text eligibility assessment, and duplicate removal, 31 studies met inclusion criteria and were retained for final synthesis (Figure 1). Of these, 25 studies were identified through PubMed and 6 additional non-duplicative studies were identified through Web of Science.

The characteristics of included studies are summarized in Table 1. The included evidence base was methodologically diverse. Twenty-two studies were classified as Primary Evidence Studies, including cross-sectional analyses, observational studies, randomized or experimental designs, qualitative studies, and workforce database analyses. Nine studies were classified as Contextual/Conceptual Articles, including systematic reviews, scoping reviews, commentaries, methodological papers, and conceptual articles relevant to concordance, discordance, implicit bias, communication, and health equity. As described in the data extraction framework, contextual and conceptual articles were used to support interpretation of the literature but were not weighted equally with primary empirical evidence when drawing conclusions regarding healthcare outcomes.

### 3.2 Communication and trust

Communication and trust were the most frequently represented domains across the included literature. Fourteen studies directly examined communication, trust, shared decision-making, implicit bias, or patient perceptions of interpersonal care (31–43). Fourteen of thirty-one included studies examined communication, trust, shared decision-making, implicit bias, or interpersonal care experiences. Eleven studies reported positive associations between concordance-related factors and communication or trust outcomes, whereas three reported mixed or attenuated findings after adjustment for contextual variables.

Several studies identified communication quality as a key mechanism. Shen et al. found that racial concordance was associated with better communication in several domains, although effects were not uniform across all measures (31). Lin and Kressin reported racial/ethnic differences in patients' experiences of treatment decision-making and physician communication (41). Assari found that psychosocial determinants of communication satisfaction differed across racially concordant and discordant patient–physician interactions (37). Hagiwara et al. and Hagiwara and Dent further emphasized that provider implicit bias and limitations in existing communication coding systems may obscure important dynamics in racially discordant encounters (66, 92).

However, findings were not uniformly positive. Sweeney et al. found that patient race appeared to explain communication ratings more strongly than concordance itself, and Miller et al. concluded that many prior studies did not demonstrate a consistent independent association between concordance and communication quality after adjustment for covariates (48). These mixed findings suggest that concordance may shape communication through complex interpersonal and structural pathways rather than through a simple direct effect.

### 3.3 Patient satisfaction, preference, and engagement

Patient satisfaction, preference, and engagement represented another major evidence domain. Nine studies examined satisfaction, patient preference, therapeutic connection, provider ratings, or engagement with care (34–36, 38, 39, 44–47). Of these nine studies, eight reported positive associations between concordance-related factors and patient satisfaction, provider ratings, therapeutic connection, or patient preference, while one study reported more nuanced findings that varied by outcome domain.

Walker et al. reported associations between racial/ethnic and sex concordance and satisfaction scores in primary care (45). Rathert et al. found that therapeutic connection varied by patient race/ethnicity, suggesting that relational quality may differ across racialized healthcare experiences (39). Crawford et al. found that racial and gender concordance influenced patient assessments of hospitalist performance (38). Moore et al. provided qualitative evidence that many Black patients preferred racially concordant providers because of perceived shared understanding, comfort, communication, and trust (44).

Saha and Beach similarly demonstrated that physician race influenced patient decision-making and physician ratings in an experimental vignette design (36). Holm et al. found that many participants considered provider similarity important, with stronger preference reported among racially and ethnically minoritized groups (44). Collectively, these findings suggest that concordance may matter not only as a demographic pairing, but as a perceived marker of understanding, respect, and relational safety (35).

### 3.4 Healthcare utilization, adherence, and costs

Six studies examined healthcare utilization, adherence, care continuity, shared decision-making, or healthcare expenditures (33, 41, 47–50). Five of the six studies reported positive associations between concordance-related factors and healthcare utilization, adherence, care continuity, shared decision-making, or healthcare expenditures, whereas one study demonstrated more limited or context-dependent effects. Jetty et al. found that patient–physician racial concordance was associated with improved healthcare use and lower healthcare expenditures among minority populations (52). Ma et al. reported that patient–provider race/ethnicity concordance was associated with provider visit patterns (47). Ma et al. later examined racial disparities in medication adherence and the patient–provider relationship, further supporting the relevance of concordance-related relational factors in chronic disease management (50).

Thomas et al. examined shared decision-making among Black patients at risk for sudden cardiac arrest, reinforcing the importance of culturally responsive decision support in high-stakes clinical contexts (32). Charlot et al. found that patient-navigator race and language concordance influenced care after abnormal cancer screening results (55). Together, these studies suggest that concordance-related factors may influence whether patients access, continue, and act upon healthcare recommendations.

### 3.5 Clinical outcomes

Eight studies addressed clinical outcomes or specialty-specific healthcare outcomes, including pain, surgery, diabetes care, dermatology, maternal/infant health, addiction treatment, and cancer-related follow-up (46, 49, 51–56). Six of the eight studies reported favorable associations between concordance-related factors and clinical or specialty-specific outcomes, while two studies concluded that evidence remained limited or insufficient to support definitive conclusions. Anderson et al. found that clinician–patient racial/ethnic concordance influenced pain-related outcomes in simulated clinical interactions (31). Suleiman et al. reported that patient–physician racial concordance increased the likelihood of total knee arthroplasty recommendation (51). Shannon et al. found associations between patient–surgeon racial/ethnic concordance and outcomes among older adults undergoing surgery (37).

In chronic disease care, Kindratt et al. examined racial/ethnic and gender concordance among women of childbearing age with diabetes (53). In addiction treatment, Beaugard et al. found limited but important evidence suggesting that racial concordance may improve engagement and treatment experiences for Black patients (53). Johnson argued that racial concordance may be relevant to strategies aimed at protecting Black mothers, children, and infants (46). These findings suggest that concordance may have clinical relevance in selected settings, although the evidence remains heterogeneous and outcome-specific.

### 3.6 Workforce representation and structural access

Workforce representation emerged as a structural theme rather than a purely interpersonal one. Three studies directly examined workforce representation or structural access to concordant care, all of which identified persistent underrepresentation of Black physicians as a potential barrier to patient access to concordant care. Two included studies, together with national workforce data directly addressed physician workforce composition or access to concordant care (57–59). Kim et al. demonstrated variation in racial and ethnic representation among physicians in U.S. internal medicine residency programs (39). Kirksey et al. documented underrepresentation of Black physicians in vascular surgery (58). These studies suggest that even when concordant care is preferred or potentially beneficial, structural workforce constraints may limit patient access to racially/ethnically concordant clinicians.

This theme is important because concordance cannot be implemented as a patient-centered option unless the physician workforce includes adequate representation. Therefore, workforce diversity may function as an upstream determinant of access to concordant care.

### 3.7 Study quality, heterogeneity, and summary of evidence

Study quality varied across the included literature (Table 2). Most primary empirical studies were rated as moderate to high quality, with strengths including large sample sizes, nationally representative datasets, validated outcome measures, and robust statistical adjustment. However, most studies remained observational or cross-sectional in design, limiting causal inference. Several studies relied primarily on patient-reported outcomes, while a small number of included articles were conceptual or commentary-based and therefore provided contextual rather than empirical evidence. Overall, the evidence base was judged to be moderate in quality, supporting cautious interpretation of concordance-related associations while highlighting the need for additional prospective and experimental research.

No clear evidence of systematic reporting bias was identified during narrative synthesis; however, publication bias cannot be excluded because formal publication-bias assessment methods were not performed.

Among the twenty-two primary empirical studies, seventeen reported predominantly favorable associations between concordance-related factors and healthcare communication, trust, satisfaction, engagement, utilization, adherence, or clinical outcomes. Five studies reported mixed, indirect, attenuated, or context-dependent findings. Across all thirty-one included studies, Communication and trust represented the most frequently examined domains (14/31, 45.2%), followed by patient satisfaction and engagement (9/31, 29.0%), clinical outcomes (8/31, 25.8%), healthcare utilization/adherence/costs (6/31, 19.4%), and workforce representation/structural access (3/31, 9.7%).

Importantly, few studies directly differentiated among Black subgroups such as Foundational Black Americans, African immigrants, and Afro-Caribbean populations. As a result, subgroup-specific conclusions remain limited. The available literature supports the relevance of concordance for Black populations broadly, but it does not yet provide sufficient evidence to determine whether concordance-related effects differ across distinct Black subgroups. Future studies should incorporate more refined subgroup characterization where appropriate.

Taken together, the included evidence suggests that physician–patient racial/ethnic concordance is frequently associated with improved healthcare communication, trust, satisfaction, engagement, utilization, and selected clinical outcomes. However, these associations are not uniform across all settings, and concordance should be interpreted as one potentially meaningful factor within a broader framework that includes cultural responsiveness, clinician communication, implicit bias reduction, patient preference, and workforce representation.

## 4 Discussion

### 4.1 Main findings

This integrative systematic review found that physician–patient or provider–patient racial/ethnic concordance is frequently associated with improved healthcare experiences among Black and racially/ethnically minoritized patients, particularly in the domains of communication, trust, satisfaction, shared decision-making, healthcare utilization, and perceived quality of care. Across the included studies, the strongest and most consistent evidence related to patient experience and interpersonal care rather than uniformly measurable clinical outcomes. Concordance was associated with improved satisfaction in primary care, greater perceived provider similarity, stronger preference for racially concordant providers, improved healthcare utilization, lower expenditures, and more favorable patient-provider relationship measures (34, 35, 39, 44, 45, 47, 48, 60).

The evidence also suggests that concordance may have relevance beyond routine outpatient care. Included studies linked concordance-related factors to pain perception, cancer navigation, surgical recommendations, surgical outcomes, diabetes care, medication adherence, addiction treatment, and maternal/infant health discourse (49–56). However, these findings should not be interpreted as uniformly causal. Much of the evidence base is observational, cross-sectional, or based on self-reported outcomes, and the magnitude and consistency of concordance effects varied across settings.

### 4.2 Mechanisms: trust, communication, and engagement

The most plausible mechanisms linking concordance to healthcare outcomes appear to involve trust, communication quality, patient comfort, perceived respect, shared decision-making, and engagement. Several studies suggest that concordance may improve the interpersonal conditions under which patients disclose concerns, ask questions, evaluate recommendations, and remain engaged in care (31, 34, 35, 37, 39, 41).

At the same time, the literature does not support a simplistic conclusion that concordance automatically improves every communication outcome. Sweeney et al. found that patient race explained communication ratings more strongly than concordance itself, while Miller et al. reported mixed evidence regarding the independent relationship between concordance and physician–patient communication after adjustment for covariates (41, 48). These mixed findings are important because they suggest that concordance may operate through broader relational and structural pathways rather than through demographic matching alone.

Implicit bias and discordant communication dynamics may also help explain why concordance is sometimes meaningful. Prior work indicates that clinicians' implicit racial attitudes can affect medical visit communication and patient ratings of interpersonal care (12). Hagiwara et al., Hall et al., and Hagiwara and Dent further suggest that implicit bias, communication behaviors, and limitations in current coding systems may obscure important features of racially discordant clinical encounters (60, 91, 92). In this context, concordance should be understood as one potentially important contributor to patient-centered care, not as a substitute for cultural humility, bias reduction, and high-quality communication training.

### 4.3 Workforce implications

The findings also have implications for workforce representation and structural access to concordant care. Even where patients prefer concordant providers or where concordance is associated with improved care experiences, access to concordant care depends on the composition and distribution of the healthcare workforce. Prior national workforce data show persistent underrepresentation of Black physicians, and included studies further demonstrate representation gaps within residency programs and specialty workforces (57–59).

This matters because concordance cannot function as a patient-centered option if the workforce does not include sufficient representation. Workforce diversification, recruitment, retention, mentorship, and equitable training pathways should therefore be understood as health systems interventions with potential downstream relevance for patient trust, access, and care engagement. At the same time, workforce diversification should complement—not replace—system-wide investment in culturally responsive care, communication quality, and institutional accountability (61–63).

### 4.4 Heterogeneity within black populations

A major interpretive issue is that most included concordance studies analyzed Black patients as a broad aggregate category rather than differentiating among Foundational Black Americans, African immigrants, Afro-Caribbean immigrants, and other Black subgroups. This limits the ability to draw subgroup-specific conclusions. The present review therefore does not demonstrate that concordance effects are unique to FBAs, nor does it establish that FBAs experience concordance differently from other Black populations. Consequently, references to Foundational Black Americans throughout this review should be interpreted primarily as a rationale for subgroup disaggregation and future investigation rather than as evidence that currently observed concordance effects are specific to FBAs. The present evidence base supports conclusions regarding Black populations broadly but remains insufficient to support definitive subgroup-specific inferences.

Rather, the findings suggest that future concordance research may benefit from more refined subgroup characterization within Black populations, as current aggregation practices may obscure meaningful heterogeneity in healthcare experiences, institutional trust, communication expectations, and outcomes. Prior literature shows that Black subgroups in the United States differ in social determinants of health, cardiometabolic risk, cancer mortality, migration history, and healthcare system distrust (15–20, 23, 30, 64). These differences provide a rationale for future studies to examine whether nativity, parental birthplace, lineage, migration history, or other subgroup measures modify concordance-related associations.

## 5 Limitations

This review has several limitations. First, although the revised search strategy included both PubMed and Web of Science, relevant studies indexed in other databases such as Embase, Scopus, CINAHL, PsycINFO, or Sociological Abstracts may not have been captured. Second, the search was limited to studies published from 2015 to 2026, which emphasized contemporary literature but may have excluded older foundational studies.

Third, the included literature was methodologically heterogeneous. Studies varied in design, population, clinical setting, exposure definition, and outcome measurement, which precluded meta-analysis. Most primary empirical studies were observational, cross-sectional, or survey-based, limiting causal inference. Several relied on patient-reported outcomes, which are highly relevant to patient experience but may be influenced by unmeasured contextual factors.

Fourth, a formal risk-of-bias assessment and GRADE certainty evaluation were not performed. Instead, evidence was interpreted narratively with attention to design limitations, consistency, directness, and relevance. Fifth, some included studies examined concordance indirectly through related constructs such as implicit bias, patient preference, therapeutic connection, or workforce representation. These studies were useful for conceptual synthesis but should not be interpreted as direct tests of concordance effects.

Finally, very few included studies differentiated among Black subgroups. As a result, subgroup-specific inference for Foundational Black Americans, African immigrants, and Afro-Caribbean immigrants remains limited. Future research should incorporate more precise subgroup measures where appropriate and avoid treating Black populations as analytically homogeneous.

## 6 Conclusion

Physician–patient racial/ethnic concordance appears to be associated with improved healthcare experiences across several domains, particularly communication, trust, satisfaction, shared decision-making, patient engagement, healthcare utilization, and perceived quality of care. Evidence for clinical outcomes is promising in selected settings but remains less consistent and more context-dependent.

The findings support a balanced interpretation: concordance is not a universal solution and should not be treated as a substitute for high-quality care, cultural responsiveness, or bias reduction. Instead, concordance may function as one meaningful interpersonal and structural factor within a broader health equity framework. Strengthening workforce representation, improving communication training, reducing implicit bias, and expanding culturally responsive care may work together to improve healthcare experiences for Black patients and other underserved populations.

Future research should use more rigorous designs, clearer outcome definitions, and more refined subgroup characterization within Black populations. In particular, studies should examine whether concordance-related effects differ by nativity, migration history, lineage, or other markers of within-group heterogeneity. This would allow future work to better determine how concordance, cultural responsiveness, and workforce representation can be implemented in ways that are evidence-based, patient-centered, and responsive to the diversity of Black populations in the United States.

## Data Availability

The original contributions presented in the study are included in the article/Supplementary material, further inquiries can be directed to the corresponding author.
