# Supplementary material for: Exploring the role of racial/ethnic patient–physician concordance in optimizing health and patient outcomes among black populations in the United States: an integrative systematic review
Source: Front Public Health. 2026 Jun 24;14:1838171. doi: 10.3389/fpubh.2026.1838171 (PMC13341696; doi:10.3389/fpubh.2026.1838171)
Supplement: Supplementary file 1 [file Table_1.docx]

| **Database / Source** | **Search Label** | **Search Query** | **Date of Search** | **Inclusion Filters Applied at Screening** | **Exclusion Filters Applied at Screening** | **Purpose** |
| --- | --- | --- | --- | --- | --- | --- |
| PubMed | Search 1 | (“racial concordance” OR “ethnic concordance” OR “race concordance” OR “patient physician concordance” OR “patient provider concordance”) AND (“Black patients” OR “African American” OR minority) AND (trust OR communication OR satisfaction OR adherence OR utilization OR outcomes OR mortality OR “shared decision making”) | May 22, 2026 | Published 2015–2026; examined physician–patient or provider–patient racial/ethnic concordance; addressed communication, trust, satisfaction, shared decision-making, adherence, utilization, clinical outcomes, or workforce-related access; U.S.-based populations or healthcare settings relevant to Black populations | Studies published before 2015; non-U.S. populations; language concordance only; conference abstracts without sufficient methodological detail; studies not examining racial/ethnic concordance or concordance-related mechanisms; studies focused solely on broad disparities without relevance to concordance | Identify contemporary empirical and conceptual literature examining physician–patient racial/ethnic concordance and healthcare outcomes among Black populations in the United States |
| Web of Science | Search 2 | (“racial concordance” OR “ethnic concordance” OR “race concordance” OR “patient physician concordance” OR “patient provider concordance”) AND (“Black patients” OR “African American” OR minority) AND (trust OR communication OR satisfaction OR adherence OR utilization OR outcomes OR mortality OR “shared decision making”) | May 22, 2026 | Published 2015–2026; examined physician–patient or provider–patient racial/ethnic concordance; addressed communication, trust, satisfaction, shared decision-making, adherence, utilization, clinical outcomes, or workforce-related access; U.S.-based populations or healthcare settings relevant to Black populations | Studies published before 2015; non-U.S. populations; language concordance only; conference abstracts without sufficient methodological detail; studies not examining racial/ethnic concordance or concordance-related mechanisms; studies focused solely on broad disparities without relevance to concordance | Supplement PubMed results and capture additional interdisciplinary literature not indexed in PubMed regarding concordance, healthcare experiences, and health outcomes among Black populations |

**Supplementary Table S1. Literature Search Strategy and Screening Criteria.** Structured searches were conducted in PubMed and Web of Science on May 22, 2026. Searches were limited to studies published between January 1, 2015 and May 22, 2026. Articles were screened according to predefined eligibility criteria developed from the review question and PICOS framework. The final review included 31 studies after screening and duplicate removal.
