# Supplementary material for: Exploring the role of racial/ethnic patient–physician concordance in optimizing health and patient outcomes among black populations in the United States: an integrative systematic review
Source: Front Public Health. 2026 Jun 24;14:1838171. doi: 10.3389/fpubh.2026.1838171 (PMC13341696; doi:10.3389/fpubh.2026.1838171)
Supplement: Supplementary file 2 [file Table_2.docx]

**PRISMA 2020 CHECKLIST**

| **Section/Topic** | **Item** | **PRISMA 2020 Checklist Item** | **Location in Manuscript** |
| --- | --- | --- | --- |
| TITLE | 1 | Identify the report as a systematic review | Title Page |
| ABSTRACT | 2 | See PRISMA 2020 for Abstracts checklist | Abstract |
| INTRODUCTION | 3 | Describe rationale for review | Introduction – Sections 1.1–1.4 |
| INTRODUCTION | 4 | State objectives/questions | Introduction – Section 1.1 |
| METHODS | 5 | Eligibility criteria | Methods – Section 2.2 Eligibility Criteria |
| METHODS | 6 | Information sources | Methods – Section 2.1 Data Sources and Search Strategy |
| METHODS | 7 | Search strategy | Methods – Section 2.1 Data Sources and Search Strategy |
| METHODS | 8 | Selection process | Methods – Section 2.4 Study Selection |
| METHODS | 9 | Data collection process | Methods – Section 2.5 Data Extraction |
| METHODS | 10a | Outcomes sought | Methods – Sections 2.3 PICOS Framework and 2.5 Data Extraction |
| METHODS | 10b | Other variables collected | Methods – Section 2.5 Data Extraction |
| METHODS | 11 | Risk of bias assessment | Methods – Sections 2.7 Risk of Bias and Certainty of Evidence and 2.8 Study Quality Assessment |
| METHODS | 12 | Effect measures | Not applicable (no meta-analysis performed) |
| METHODS | 13a | Synthesis eligibility decisions | Methods – Sections 2.5 and 2.6 |
| METHODS | 13b | Data preparation methods | Methods – Section 2.5 Data Extraction |
| METHODS | 13c | Methods used to tabulate/display results | Table 1; Table 2; Figure 1; Methods – Section 2.6 |
| METHODS | 13d | Synthesis methods and rationale | Methods – Section 2.6 Data Synthesis |
| METHODS | 13e | Exploration of heterogeneity | Methods – Section 2.6; Discussion – Section 4.4 |
| METHODS | 13f | Sensitivity analyses | Not performed |
| METHODS | 14 | Reporting bias assessment | Methods – Section 2.7 Risk of Bias and Certainty of Evidence |
| METHODS | 15 | Certainty assessment | Methods – Sections 2.7 and 2.8 |
| RESULTS | 16a | Study selection results | Results – Section 3.1 Study Selection and Descriptive Characteristics |
| RESULTS | 16b | Excluded studies and reasons | Supplementary Materials; Data Availability Statement |
| RESULTS | 17 | Study characteristics | Results – Section 3.1; Table 1 |
| RESULTS | 18 | Risk of bias in studies | Results – Section 3.7; Table 2 |
| RESULTS | 19 | Results of individual studies | Table 1 |
| RESULTS | 20a | Characteristics and risk of bias contributing to synthesis | Results – Section 3.7 |
| RESULTS | 20b | Results of syntheses | Results – Sections 3.2–3.7 |
| RESULTS | 20c | Investigation of heterogeneity | Results – Sections 3.2–3.7; Discussion – Section 4.4 |
| RESULTS | 20d | Sensitivity analyses | Not performed |
| RESULTS | 21 | Reporting biases | Results – Section 3.7 |
| RESULTS | 22 | Certainty of evidence | Results – Section 3.7 |
| DISCUSSION | 23a | General interpretation of results | Discussion – Sections 4.1–4.4 |
| DISCUSSION | 23b | Limitations of evidence | Discussion – Section 5 Limitations |
| DISCUSSION | 23c | Limitations of review processes | Discussion – Section 5 Limitations |
| DISCUSSION | 23d | Implications for practice, policy, and future research | Discussion – Sections 4.3, 4.4, and 6 Conclusion |
| OTHER INFORMATION | 24a | Registration information | Methods – Section 2.1 Data Sources and Search Strategy |
| OTHER INFORMATION | 24b | Protocol access | Methods – Section 2.1 Data Sources and Search Strategy |
| OTHER INFORMATION | 24c | Amendments to protocol | Not applicable (no registered protocol) |
| OTHER INFORMATION | 25 | Sources of support | Funding |
| OTHER INFORMATION | 26 | Competing interests | Conflict of Interest |
| OTHER INFORMATION | 27 | Availability of data, code, and other materials | Data Availability Statement |

**Table S2. PRISMA 2020 Checklist and Manuscript Location of Reported Items.**
The Preferred Reporting Items for Systematic Reviews and Meta-Analyses (PRISMA 2020) checklist was used to evaluate reporting completeness. The table identifies the manuscript pages, sections, and line ranges corresponding to each PRISMA reporting criterion addressed in this integrative systematic review.
